# Investigating racial/ethnic differences in procedure experience in obstetrics & gynecology trainees at a single academic institution: a retrospective cohort study

**DOI:** 10.1186/s12909-024-05363-9

**Published:** 2024-05-23

**Authors:** Patricia GiglioAyers, Christine E. Foley, Beth Cronin, Dayna Burrell

**Affiliations:** 1 Department of Obstetrics and Gynecology, Women and Infants Hospital, 101 Dudley St, 02905 Providence, RI USA; 2 The Warren Alpert Medical School of Brown University, 222 Richmond Street, 02903 Providence, RI USA

**Keywords:** Race/Ethnicity, Residency training, Surgical education, Obstetrics and gynecology, Operative logs

## Abstract

**Background:**

Discrimination is common in medical education. Resident physicians of races and ethnicities underrepresented in medicine experience daily discrimination which has been proven to negatively impact training. There is limited data on the impact of resident race/ethnicity on OB/GYN surgical training. The objective of this study was to investigate the impact of race/ethnicity on procedural experience in OB/GYN training.

**Methods:**

A retrospective analysis of graduated OB/GYN resident case logs from 2009 to 2019 was performed at a single urban academic institution. Self-reported race/ethnicity data was collected. Association between URM and non-URM were analyzed using t-tests. Trainees were categorized by self-reported race/ethnicity into underrepresented in medicine (URM) (Black, Hispanic, Native American) and non-URM (White, Asian).

**Results:**

The cohort consisted of 84 residents: 19% URM (*N* = 16) and 79% non-URM (*n* = 66). Difference between URM and non-URM status and average case volume was analyzed using t-tests. There was no difference between non-URM and URM trainees and reported mean number of Total GYN (349 vs. 334, *p* = 0.31) and Total OB (624 vs. 597, *P* = 0.11) case logs. However, compared with non-URM, on average URM performed fewer Total procedures (1562 vs. 1469, *P* = 0.04). Analyzing individual procedures showed a difference in average number of abortions performed between URM and non-URM (76 vs. 53, *P* = 0.02). There were no other statistically significant differences between the two groups.

**Conclusions:**

This single institution study highlights potential differences in trainee experience by race/ethnicity. Larger national studies are warranted to further explore these differences to identify bias and discrimination, and to ensure equitable experience for all trainees.

## Introduction

Discrimination is common in medical education, with nearly 60% of medical trainees experiencing at least one form of harassment or discrimination during their training [1]. Race/ethnicity has been proven to negatively impact medical student experiences and evaluations [2, 3]. Although data remains limited, a rising number of studies explore the impact of race/ethnicity on residency training.

Resident physicians of races & ethnicities underrepresented in medicine endure daily microaggressions and biases [4]. In general surgery, up to 24% of residents report experiencing discrimination based on race/ethnicity or religion, with highest rates (70%) reported among Black residents [5–7]. Black surgical residents are 4.2 times more likely to experience high levels of perceived daily discrimination [7]. Discriminatory acts include being mistaken for another person of the same race, mistaken for nonphysicians, and experiencing different standards of evaluation [5]. Compared with their White counterparts, non-White residents experience increase feelings of isolation and judgement [8]. Surgical residents who experience discrimination also reported higher rates of burnout, thoughts of attrition, and suicidal thoughts [5, 6]. A recent study investigating the relationship between gender, race/ethnicity and general surgery resident case volume cites a correlation between racial/ethnic categories underrepresented in medicine (URM) (identified as Black, Hispanic or Native American) and lower operative volumes at graduation [9].

Data regarding the impact of race/ethnicity on training in Obstetrics and Gynecology (OB/GYN) is limited. OB/GYN is reported to have the highest percentage of trainees from racial and ethnic backgrounds underrepresented in medicine at 19% among the surgical subspecialties [10]. However, recent data from 2022 demonstrated there is a greater proportion of White physicians at the fellowship level compared to residency level [11]. This trend persists in academic medicine, with a higher proportion of white physicians in leadership positions and with higher academic ranks [12]. Despite multiple initiatives by national organizations within OB/GYN to address racial and ethnic disparities [13, 14], studies exploring racial disparities and discrimination are sparse in OB/GYN literature. To the authors knowledge, there is no published data on the impact of race/ethnicity on resident surgical training in OB/GYN. Specifically, there is no data on the impact of race on the fundamental metric of surgical volume during gynecology residency training. The aim of this study was to begin by exploring the impact of race/ethnicity on OB/GYN procedural experience in residency training at a single institution.

## Methods

A retrospective analysis of graduated OB/GYN resident procedural case logs per the Accreditation Council for Graduate Medical Education (ACGME) from 2009 to 2019 at a single institution was performed. The research was deemed exempt by the IRB and was determined to be non-human subjects research. Self-reported race/ethnicity as limited by ERAS check boxes was collected. Trainees were categorized into URM (Black, Hispanic, Native American) and non-URM (White, Asian). The institution instructs residents to log a procedure if active participation as the primary surgeon is > 50% of the procedure. The primary outcome was total number of surgical procedures logged by a graduating resident. Secondary outcomes included procedure logs for the following ACGME categories: Normal spontaneous vaginal delivery (NSVD), Cesarean section (CS), Operative delivery (ODEL), Abdominal hysterectomy (AHYST), Vaginal hysterectomy (VHYST), Laparoscopic hysterectomy (LHYST), Minimally Invasive Hysterectomy (MIH), Total Hysterectomy (THYST), Incontinence and pelvic floor (ISPF), Laparoscopy (LAPS), Operative Hysteroscopy (OHYST), Abortion (ABORT), Transvaginal ultrasound (TVUS), Surgery for invasive cancer (SIC). Total numbers of cases, total obstetric (Total OB: CS, NSVD, ODEL), and total gynecologic (Total GYN: THYST, LAPS, OHYST) cases were collected. Residents in OB/GYN who completed the four-year residency training program were included in the analysis. Trainees who transferred training programs during residency or did not complete residency were excluded. Procedures were reported as mean number of procedures per ACGME category per group (URM vs. non-URM). Differences between URM and non-URM status and mean case volumes were analyzed using t-tests.

## Results

The cohort consisted of 84 residents. Residents who self-selected the ACGME category of “none of the above” (*n* = 2) were excluded from the URM vs. non-URM analyses. There was a total of 82 residents included in the final analysis: 66 non-URM (78.57%). (Table 1) There were no differences between non-URM and URM trainees and reported mean number of Total GYN (349 vs. 334, *p* = 0.31) and Total OB (624 vs. 597, *P* = 0.11) case logs. However, URM trainees had significantly fewer Total procedures (1469 vs. 1562, *P* = 0.04) than their non-URM counterparts (Table 2). Analyzing specific procedures showed when comparing mean number of abortions, URM trainees experienced significantly less abortions (76 vs. 53, *P* = 0.02) than non-URM trainees. No differences were found between non-URM and URM trainees in all other specific individual procedure categories (Table 2).

**Table 1:** Demographic characteristics of residents graduating from 2009 to 2019

Race/Ethnicity	Total N =	%
**URM**	16	19.1
American Indian or Alaskan Native	1	1.2
Black/African American	5	6.0
Hispanic/Latino	10	11.9
**Non-URM (Asian, White)**	66	78.6
Asian	8	9.5
White	58	69.1
**Other**		
None of the above*	2	2.4

*excluded from URM vs. Non-URM analyses.

*Abbreviations: Underrepresented in medicine (URM).*

**Table 2:** OB/GYN Resident procedural experience, non URM versus URM.

Procedure		N	Mean	Standard Deviation	[95% conf interval]		p-value	
Total cases:		non-URM	66	1,562.2	± 156.9	1,523.7	- 1,600.8	*0.04
		URM	16	1,468.9	± 176.4	1,374.9	- 1,562.9	
Total OB	non-URM	66	624.4	± 62.0	609.1	- 639.6	0.11	
	URM	16	596.6	± 58.3	565.6	- 627.7		
NSVD		66	271.9	± 45.4	260.8	- 283.1	0.42	
		16	261.4	± 51.4	234.0	- 288.8		
CS		66	326.2	± 37.1	317.1	- 335.3	0.14	
		16	310.3	± 42.1	287.9	- 332.8		
ODLE		66	26.2	± 7.2	24.5	- 28.0	0.52	
		16	24.9	± 6.4	21.6	- 28.3		
Total GYN	non-URM	66	349.2	± 51.4	336.6	- 361.9	0.31	
	URM	16	334.8	± 47.3	309.6	- 360.0		
THYST		66	135.9	± 16.7	131.8	- 140.0	0.59	
		16	133.4	± 17.8	123.9	- 142.8		
LAPS		66	122.9	± 30.0	115.6	- 130.3	0.29	
		16	114.4	± 21.5	102.9	- 125.8		
OHYST		66	90.4	± 20.7	85.3	- 95.5	0.58	
		16	87.1	± 23.9	74.3	- 99.8		
Other:								
AHYST	non-URM	66	51.0	± 15.6	47.2	- 54.8	0.19	
	URM	16	45.3	± 16.3	36.6	- 54.0		
VHYST		66	30.9	± 6.5	29.3	- 32.5	0.88	
		16	30.6	± 9.2	25.7	- 35.6		
LHYST		66	54.0	± 19.0	49.3	- 58.7	0.50	
		16	57.5	± 16.5	48.7	- 66.3		
MIH		66	84.9	± 20.6	79.9	- 90.0	0.56	
		16	88.1	± 14.3	80.5	- 95.8		
ISPF		66	77.4	± 25.8	71.0	- 83.7	0.87	
		16	78.7	± 35.9	59.6	- 97.8		
ABORT		66	76.3	± 35.4	67.6	- 85.0	*0.02	
		16	53.5	± 25.8	39.8	- 67.3		
TVUS		66	128.7	± 78.0	109.5	- 147.8	0.07	
		16	104.1	± 37.6	84.0	- 124.1		
SIC		66	85.5	± 28.1	78.6	- 92.4	0.45	
		16	79.7	± 25.9	65.9	- 93.5		

*p-value < 0.05. Abbreviations: Underrepresented in medicine (URM), Normal spontaneous vaginal delivery (NSVD), Cesarean section (CS), Operative delivery (ODEL), Total Hysterectomy (THYST), Laparoscopy (LAPS), Operative Hysteroscopy (OHYST), Abdominal hysterectomy (AHYST), Vaginal hysterectomy (VHYST), Laparoscopic hysterectomy (LHYST), Minimally Invasive Hysterectomy (MIH), Incontinence and pelvic floor (ISPF), Abortion (ABORT), Transvaginal ultrasound (TVUS), Surgery for invasive cancer (SIC)

## Discussion

Resident trainees from races and ethnicities underrepresented in medicine experience daily discrimination, however there is limited data on the impact of racial/ethnic discrimination on training and postgraduate experience within OB/GYN. The importance of identifying and addressing racial and ethnic disparities within OB/GYN and medical education is widely accepted. In 2021, the ACGME launched ACGME Equity Matters, an initiative focused on learning and improvement in areas of diversity, equity and incision, and antiracism practices [13]. In 2020 ACOG, along with leading national and international women’s health organizations, released a joint statement, “Collective Action Addressing Racism.” [14] This statement specifically cites commitment to education, recognition, and scholarship as ways to eliminate inequalities in women’s health. Despite these initiatives, published research is limited.

This single institution study highlights potential differences in trainee experience by race/ethnicity and calls for further review at training programs across our specialty. This study showed a difference in total procedure experience between URM and non-URM OB/GYN residents during the 10-year time period examined. These differences may suggest discriminatory practices which are limiting procedural experience for URM residents. These findings are similar to recently published data that demonstrated a correlation between general surgery residents underrepresented in medicine or who identified as female, and lower operative volumes at graduation [9]. 

Additionally, this study observed a significant difference in the number of abortion procedures logged by URM versus non-URM trainees. In our institution, trainees have the choice to opt out of abortion procedures. This choice is not recorded as a part of the operative log but may confound this particular data point. We are unaware of any correlation between a trainee’s self-identified race and choice to perform abortion procedures. Additional work is needed to evaluate the demonstrated differences on a qualitative level to better identify the root cause(s) of the variation demonstrated, including possible sociocultural influences. Further work must be done to identify unconscious and overt biases and address discrimination to ensure all residents, regardless of race/ethnicity or gender, have an equitable training experience.

This small, single institution study calls for further review of racial and ethnic differences in procedural experience at training programs across our specialty. Although OB/GYN does have the highest percent of URM trainees among the surgical subspecialties, the lower proportion of URM physicians in fellowships and in higher academic rank positions suggests persistent institutional and structural racism. Procedural case logs are an objective and nationally utilized measure which could be further analyzed to identify and ultimately address training differences. If publicly available, these case logs could hold programs accountable for ensuring equitable procedural experience. Addressing any identified differences would not only improve resident experience and skill, but also contribute to the goal of creating a racially and ethnically diverse workforce to improve patient care in OB/GYN.

There are several limitations to this study, including variation in the accuracy and reporting practices of resident procedure logs which may impact data. Although criteria at this institution exist instructing residents to log only procedures which they performed > 50% of as the primary surgeon, residents are individually responsible for tracking and logging procedures. Furthermore, the small sample size of this study at a single institution, coupled with the variation in resident surgical experience and reporting practices between OB/GYN programs nationally, prevent this study from generalizability to all OB/GYN residency programs. This study analyzes total case logs at time of graduation, and therefore does not explore how race/ethnicity may impact procedural experience across the four years of residency and does not account for variation in logging during different times of residency. The authors also recognize that increased procedural numbers do not necessarily translate to procedural competency. Although differences may suggest training inequity among URM vs. non-URM residents, variation in procedural numbers may not reflect trainee competency at time of graduation.

## Conclusions

Differences may exist in Obstetrics and Gynecology procedural experience by trainee race/ethnicity. Larger national studies are warranted to further explore these differences to identify bias and discrimination, and to ensure equitable experience for all trainees.

## Data Availability

The datasets used and/or analyzed during the current study are available from the corresponding author on reasonable request.

## References

[1] Fnais N, Soobiah C, Chen MH (2014). Harassment and discrimination in medical training: a systematic review and meta-analysis. Acad Med.

[2] Woolf K (2020). Differential attainment in medical education and training. BMJ.

[3] Orom H, Semalulu T, Underwood W (2013). The social and learning environments experienced by underrepresented minority medical students: a narrative review. Acad Med.

[4] Osseo-Asare A, Balasuriya L, Huot SJ (2018). Minority Resident Physicians’ views on the role of Race/Ethnicity in their training experiences in the Workplace. JAMA Netw Open.

[5] Yuce TK, Turner PL, Glass C (2020). National Evaluation of Racial/Ethnic Discrimination in US Surgical Residency Programs. JAMA Surg.

[6] Hu YY, Ellis RJ, Hewitt DB (2019). Discrimination, abuse, harassment, and Burnout in Surgical Residency Training. N Engl J Med.

[7] Khubchandani JA, Atkinson RB, Ortega G (2022). Perceived discrimination among Surgical residents at Academic Medical centers. J Surg Res.

[8] Wong RL, Sullivan MC, Yeo HL, Roman SA, Bell RH, Sosa JA (2013). Race and surgical residency: results from a national survey of 4339 US general surgery residents. Ann Surg.

[9] Eruchalu CN, He K, Etheridge JC (2022). Gender and Racial/Ethnic disparities in operative volumes of graduating general surgery residents. J Surg Res.

[10] Nieblas-Bedolla E, Williams JR, Christophers B, Kweon CY, Williams EJ, Jimenez N (2020). Trends in Race/Ethnicity among applicants and matriculants to US Surgical specialties, 2010–2018. JAMA Netw Open.

[11] Talbott JMV, Wasson MN (2022). Sex and Racial/Ethnic Diversity in Accredited Obstetrics and Gynecology Specialty and Subspecialty Training in the United States. J Surg Educ.

[12] Wooding DJ, Das P, Tiwana S, Siddiqi J, Khosa F (2020). Race, ethnicity, and gender in academic obstetrics and gynecology: 12-year trends. Am J Obstet Gynecol MFM.

[13] Diversity E, Inclusion. Accessed December 6, 2022. https://www.acgme.org/what-we-do/diversity-equity-and-inclusion/.

[14] Joint Statement: Collective Action Addressing Racism. Accessed December 6. 2022. https://www.acog.org/news/news-articles/2020/08/joint-statement-obstetrics-and-gynecology-collective-action-addressing-racism.

